# Influence of Early and Regular Dental Visits on Dental Health Care Costs of Primary School Children in Amsterdam

**DOI:** 10.1016/j.identj.2025.100839

**Published:** 2025-05-31

**Authors:** Tessa S. van Ligten, Denise Duijster, Egija Zaura, Catherine M.C. Volgenant

**Affiliations:** a Department of Cariology, Academic Centre for Dentistry Amsterdam (ACTA), University of Amsterdam and Vrije Universiteit, Amsterdam, The Netherlands; b Department of Oral Public Health, Academic Centre for Dentistry Amsterdam (ACTA), University of Amsterdam and Vrije Universiteit, Amsterdam, The Netherlands; c Department of Preventive Dentistry, Academic Centre for Dentistry Amsterdam (ACTA), University of Amsterdam and Vrije Universiteit, Amsterdam, The Netherlands

**Keywords:** Early and regular dental visits, Epidemiology, Oral health care consumption, Public dental health

## Abstract

**Objectives:**

To explore whether dental visits before the age of 4 years and regular dental visits were associated with incurring dental costs at age 9 (proxy for a dental visit), and if so, what were the dental costs for primary school children in Amsterdam associated with those visits.

**Methods:**

In this retrospective, longitudinal study, sociodemographic characteristics and dental costs between 2009 and 2017 were obtained from primary school children living in Amsterdam via Statistics Netherlands. Explanatory variables were whether children visited a dentist <4 years of age between 2009 and 2011 (yes/no) and whether children regularly visited a dentist between 2012 and 2016 (yes/no). The outcome was dental costs at age 9 in 2017 (yes/no and the amount).

**Results:**

The study population consisted of 9,519 children. Dental costs <4 years of age and consecutive dental costs were associated with incurring dental costs at age 9 (aOR 2.12 [1.83-2.45]; aOR 6.48 [5.56-7.54], respectively). For those with dental costs at age 9, dental costs <4 years of age were not associated with the amount of dental costs (mean difference [MD] 5.16 [-2.69-13.00]). For children incurring consecutive dental costs, dental costs at age 9 were higher than for those without (MD 17.52 [7.35-27.69]).

**Conclusions:**

Early and regular dental visits were associated with increased odds of visiting a dentist at age 9 years. For children who visited a dentist at age 9 years, those with early and regular dental visits incurred slightly higher dental costs 5 years later, but mean differences were small and only the latter was significant. Therefore, early or regular dental visits do not lead to lower dental costs in the future.

## Introduction

Oral disease, in particular dental caries, is the most common health condition among children worldwide. In high-income countries, the prevalence of dental caries in deciduous teeth was 38% in 2019.^1^ Although a significant decrease of 7% was reported in the European Region from 1990 onwards, the disease burden is still concerning.^1^ Dental caries is unevenly distributed across socioeconomic groups.^2^ Children from families with a lower socioeconomic position (SEP), characterised by lower income, lower parental educational levels, or a migration background, experience the majority of dental caries lesions. Consequently, these children suffer from more discomfort and pain, such as trouble with eating and sleeping, associated with a lower oral health–related quality of life.^2^^,^^3^

To achieve and maintain good oral health from an early age, it is recommended to take a child to the dentist for a first visit around the eruption of their first teeth and continue with regular check-ups throughout childhood.^4^ The interval between dental check-ups is preferably at least once a year, and the frequency is based on an individual risk assessment.^1^^,^^4^^,^^5^ Regular dental visits from an early age allow for monitoring of oral health, provision of preventive measures based on individual risk assessment, and early detection of oral disease. If indicated, therapy can be provided at an early stage to prevent further progression of disease.^6^^,^^7^

In 2018, the prevalence of dental caries among Dutch children ranged from 25% in 5-year-olds and 40% in 11-year-olds to 67% in 17-year-old children.^8^ The decline in dental caries over the past decades as a result of the use of fluoride in toothpaste^6^^,^^9^ and the launch of national preventive programs in dental practices^7^^,^^10^, ^11^, ^12^, ^13^, ^14^ seems to have come to a halt for adolescents and young adults. In addition, widening inequalities in oral health exist between social groups. More children from families with a low SEP have dental caries, compared to those from families with a high SEP (5-year-old children: 29% versus 19%; 11-year-old: 43% versus 34%, respectively). Compared to native Dutch children, the prevalence of dental caries in children with a migration background is 3 times higher for 5-year-olds and almost twice as high for 11-year-olds, in both low- and high-SEP families.^8^ Hence, preventive measures seem to not be equally effective among children from various backgrounds, and children do not equally benefit from oral health care.^2^^,^^15^

Among Dutch children, only 2.8% of 0- to 1-year-olds and 65.5% of 2- to 3-year-olds visit the dentist.^16^ The assumption is that many parents bring their children to the dentist for the first time when a child is already experiencing pain, and dental caries is often already present.^17^^,^^18^ In the Netherlands, oral health care for children until 18 years of age is covered by mandatory health insurance of the parent(s), so parents are not charged out-of-pocket payments for their children’s oral health care. Oral health care is provided by private dentists in practices or, in some larger cities, including Amsterdam, via schools. In both situations, health insurance companies reimburse the incurred dental costs.^19^ The high prevalence of dental caries among children results in major health care expenses for Dutch health insurance companies, up to 645 million euros of claims annually.^20^ Although a large share of Dutch health care costs is incurred for the treatment of oral diseases, little is known about oral health care consumption of children in relation to their oral health status.^21^ Questions that remain are: Does adherence to the recommendations for an early first dental visit and regular dental check-ups in children lead to better oral health? Are there specific risk groups of children who do not see a dentist early or regularly?

**Fig. 1:**
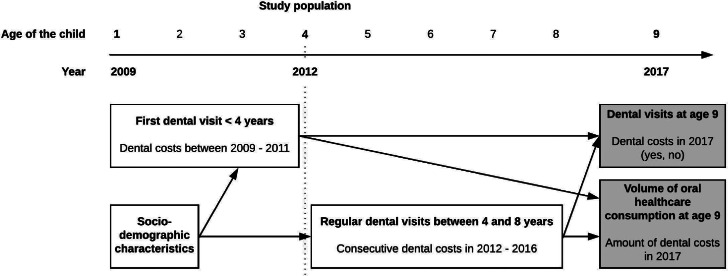
Flowchart of the design of this study.

Therefore, the aims of this study were 2-fold. First, we explored whether sociodemographic characteristics of 4-year-old children in Amsterdam were associated with the age of their first dental visit and regular dental visits throughout childhood (up to 9 years of age). Second, we assessed whether a first dental visit before the age of 4 years and regular dental visits were associated with incurring dental costs at age 9 years and, if dental costs were incurred, in what amount by that age (Figure 1).

## Methods

### Population

This study was a collaboration between the Academic Centre for Dentistry Amsterdam (ACTA) and the City of Amsterdam, the Netherlands. The study protocol was approved by the internal ethics committee of ACTA (reference number 2023-98097). The research population included all 4-year-old children who were registered in Amsterdam on January 1, 2012. Data on dental costs and sociodemographic characteristics were collected from Statistics Netherlands (CBS) from January 1, 2009, to December 31, 2017, resulting in a retrospective, longitudinal dataset of children from 1 to 9 years of age (Figure 1; the most recent data available). Children who moved away from Amsterdam between January 1, 2009, and December 31, 2017, were excluded from this study. In the Netherlands, dental costs of children are incurred through the mandatory basic medical insurance of one of their parents; in the exceptional situation when parents were not registered as insured, their children were thus not included in this study.

### Research data

The research file contained microdata from CBS, which included data on dental costs per year from 2009 to 2017. Data were obtained from a business intelligence centre which holds all health care reimbursement data of health care insurance companies in the Netherlands (Vektis). The CBS-microdata also contained data on sociodemographic characteristics of the children and their parents derived from the Dutch Personal Records Database (BRP), and from registrations such as the Education Executive Agency (DUO) of the Ministry of Education, Culture and Science, and the Tax Authorities. A unique citizen service number of each individual was used to link data on dental costs to the sociodemographic data in the research file. Since orthodontic treatment for children is not covered by basic medical insurance, those costs were not considered.

### Data on dental costs

Two dichotomous variables were used to analyse possible associations with sociodemographic characteristics and dental costs later in childhood: whether dental costs were incurred <4 years of age, and whether consecutive dental costs were incurred between the ages of 4 and 8 years. Dental costs incurred <4 years of age were defined as dental costs incurred in the years 2009-2011 (yes or no). This was a proxy variable for the first dental visit before the age of 4 years. A child was considered to have consecutive dental costs between the ages of 4 and 8 years when dental costs were incurred every year between 2012 and 2016, or when dental costs were incurred every year except for 1 year between 2012 and 2016 (versus ≥2 years with no dental costs incurred between 2012 and 2016). This was a proxy variable for regular dental visits. In the follow-up analyses, dental costs at age 9, incurred in 2017, were analysed. First, as a dichotomous outcome: dental costs at age 9, in 2017 (yes or no). Second, for those children who incurred dental costs at age 9, the total amount of dental costs incurred in 2017 (in euros) was also analysed as a continuous dependent variable (Figure 1). Low dental costs are likely indicative of dental check-ups and preventive care, while higher dental costs could indicate additional (restorative) treatment based on the cost per dental performance code.^22^

### Sociodemographic variables

Sociodemographic characteristics were split into child and parent variables. The child variables were sex, migration background, and type of household and were obtained from the BRP. Migration background was categorised as no migration background, Western migration background, and non-Western migration background. A Western migration background was defined as a country of birth of (one of) the parents in Europe, North America, Oceania, Indonesia or Japan, and a non-Western migration background applied to all other countries.^23^ Type of household was categorised as a couple of parents, single-parent household, and unknown/other. The parent variables were household income and parental educational level. Data on household income were obtained from various administrations, of which the main data suppliers were the Dutch Tax Authorities. Household income was divided into percentiles: 0-20th, 21-40th, 41-60th, 61-80th, 81-100^th^, and an unknown/other category. Parental educational level was derived from DUO and categorised as low, medium, high, and unknown/other, based on the categories in the CBS microdata.^24^

### Statistical analyses

Descriptive statistics were used to describe sociodemographic characteristics and dental costs from 2009 to 2017. First, univariable and multivariable logistic regression analyses were performed to assess associations between the sociodemographic characteristics and (1) whether dental costs were incurred <4 years of age and (2) whether consecutive dental costs were incurred from 2012 to 2016. Second, univariable and multivariable logistic regression analyses were performed to assess whether dental costs incurred <4 years of age and whether consecutive dental costs incurred between 2012 and 2016 were related to dental costs incurred at age 9, in 2017 (yes or no). Subsequently, for children who did incur dental costs at age 9, univariable and multivariable linear regression analyses were performed with the total amount of dental costs at age 9, in 2017, as the outcome (Figure 1). Since this outcome did not meet the assumption of a normal distribution because the data were skewed to the right, a natural logarithm transformation was performed, and the original and transformed data were analysed. Both analyses produced similar results, and the data proved to be robust due to the large research population. Therefore, the decision was made to present the results of the analyses with the non-transformed data, aiming to facilitate the interpretation of mean differences in euros.

The multivariable regression analyses were adjusted for the sociodemographic characteristics of sex, migration background, type of household, household income, and parental educational level. The variables were selected based on the literature, and those were entered in a single step in the analyses (Enter Method).^25^, ^26^, ^27^ An alpha of 0.05 as the threshold for statistical significance was used. All statistical analyses were conducted using IBM SPSS Statistics v25.^28^

### Missing data and data integrity

Children with missing data on dental costs between 2009 and 2017 were excluded from all analyses. Since there was a small group of children (*n* = 18) with very high dental costs in 2017 (≥1500 euros), those dental costs were also excluded to avoid distorting the analysis. Any missing values in the sociodemographic variables were categorised as ‘unknown’ and analysed as such. For most sociodemographic variables, the missing values were less than 3%, and therefore, no multiple imputation was applied.^29^^,^^30^ Small categories of sociodemographic variables were bundled in a category ‘other’, which was combined with the category ‘unknown’. Lastly, to check for possible outliers regarding dental costs, sensitivity analyses were performed with data on dental costs in 2016 and compared with data from 2017; the distribution of dental costs in both years was found to be similar.

## Results

**Fig. 2:**
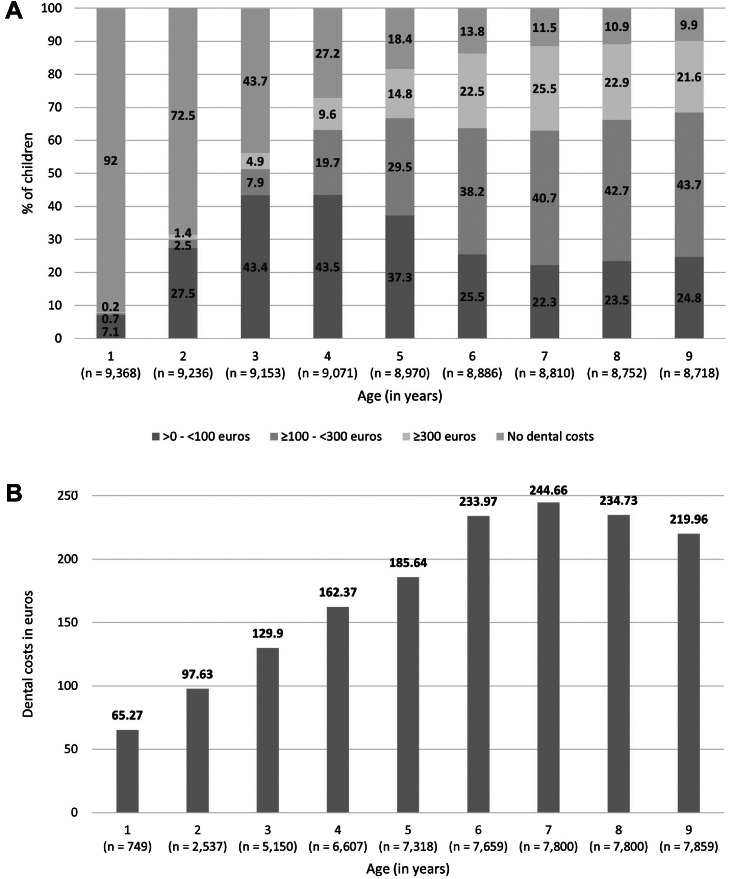
Dental costs incurred in children. A, distribution of the dental costs per age (in years), divided into no dental costs, >0 - <100 euros, ≥100 - <300 euros and ≥300 euros. B, mean dental costs in euros per age (in years) in the group of children that incurred dental costs.

The study population consisted of 9,519 4-year-old children in 2012. After excluding those with missing data on dental costs, most likely due to moving out of Amsterdam (*n* = 151 in 2009; *n* = 801 in 2017), the final study population contained 8,718 children. The proportion of children incurring dental costs increased rapidly between the ages of 1 (8.0%) and 6 (86.2%) and stabilised in consecutive years (range 88.5%-90.1%; Figure 2A). Of those who incurred dental costs, the amount was mostly low (<100 euros) for children up to the age of 5 years, while the majority of children 6 years and older incurred dental costs of 100-300 euros (Figure 2A). The mean amount of dental costs per year increased until the age of 6 (233.97 euros) and then fluctuated around that value (range 219.96-244.66 euros; Figure 2B). The study population contained slightly more boys than girls. Children with no migration background and a non-Western migration background were equally distributed; one-tenth of the study population had a Western migration background. Most children lived in a household with both parents. Regarding household income, one-third of the children were from a family in the lowest percentile, and the fewest children were from families in the highest 2 percentiles. Almost half of the parents were higher educated, and the ‘unknown/other’ category was relatively large (18%-20%; Table 1).

**Table 1:** Descriptive data of 4-year-old children in 2012, overall and categorised by dental costs <4 years of age and consecutive dental costs.

	Dental Costs <4 Years of Age			Subsequent Dental Costs		
	*N* (%)	No *n* (%)	Yes *n* (%)	*N* (%)	No *n* (%)	Yes *n* (%)
**Total**	**9,047**	**3,471**	**5,576**	**8,641**	**1,741**	**6,900**
**Child variables**						
**Sex**						
Male	4,623 (51.1)	1,796 (51.7)	2,827 (50.7)	4,412 (51.1)	943 (54.2)	3,469 (50.3)
Female	4,424 (48.9)	1,675 (48.3)	2,749 (48.9)	4,229 (48.9)	789 (45.8)	3,431 (49.7)
**Migration background**						
No migration background	3,955 (43.7)	1,438 (41.4)	2,517 (45.1)	3,902 (45.2)	861 (49.5)	3,041 (44.1)
Western migration background	1,078 (11.9)	459 (13.2)	619 (11.1)	978 (11.3)	246 (14.1)	732 (10.6)
Non-Western migration background	4,014 (44.4)	1,574 (45.3)	2,440 (43.8)	3,761 (43.5)	634 (36.4)	3,127 (45.3)
**Type of household**						
Couple of parents	7,177 (79.3)	2,605 (75.1)	4,572 (82.0)	6,879 (79.6)	1,300 (74.7)	5,579 (80.9)
Single parent	1,796 (19.9)	828 (23.9)	968 (17.4)	1,691 (19.6)	418 (24.0)	1,273 (18.4)
Unknown/other	74 (0.8)	38 (1.1)	36 (0.6)	71 (0.8)	23 (1.3)	48 (0.7)
**Parent variables**						
**Household income (percentile)**						
0-20	3,101 (34.3)	1,279 (36.8)	1,822 (32.7)	2,869 (33.2)	563 (32.3)	2,306 (33.4)
21-40	1,465 (16.2)	539 (15.5)	926 (16.6)	1,419 (16.4)	241 (13.8)	1,178 (17.1)
41-60	1,190 (13.2)	452 (13.0)	738 (13.2)	1,155 (13.4)	232 (13.3)	923 (13.4)
61-80	1,206 (13.3)	423 (12.2)	783 (14.0)	1,188 (13.7)	226 (13.0)	962 (13.9)
81-100	1,960 (21.7)	711 (20.5)	1,249 (22.4)	1,897 (22.0)	438 (25.2)	1,459 (21.1)
Unknown/other	125 (1.4)	67 (1.9)	58 (1.0)	113 (1.3)	41 (2.4)	72 (1.0)
**Educational level parent**						
Low	1,500 (16.6)	591 (17.0)	909 (16.3)	1,420 (16.4)	262 (15.0)	1,158 (16.8)
Medium	1,832 (20.2)	765 (22.0)	1,067 (19.1)	1,771 (20.5)	390 (22.4)	1,381 (20.0)
High	3,929 (43.4)	1,406 (40.5)	2,523 (45.2)	3,815 (44.1)	774 (44.5)	3,041 (44.1)
Unknown/other	1,786 (19.7)	709 (20.4)	1,077 (19.3)	1,635 (18.9)	315 (18.1)	1,320 (19.1)

Bold values indicate statistically significance.

### Sociodemographic characteristics and associations with dental costs <4 years of age and consecutive dental costs

Most sociodemographic characteristics were not significantly associated with incurring dental costs <4 years of age and with subsequent dental costs. Having a Western migration background was associated with a lower likelihood of incurring dental costs <4 years of age (adjusted odds ratio (aOR) 0.76 (0.66-0.88)) and incurring consecutive dental costs (aOR 0.83 (0.70-0.97)), compared to no migration background. Also, more children with a non-Western migration background incurred consecutive dental costs (aOR 1.39 (1.21-1.59)). Significant associations were reported for the type of household: children living with a single parent were less likely to incur dental costs <4 years of age (aOR 0.69 (0.62-0.77)) and consecutive dental costs (aOR 0.70 (0.61-0.80)), compared to children with a couple of parents. For household income, only the highest percentile was related to a lower likelihood of children incurring consecutive dental costs (aOR 0.80 (0.67-0.96)), and the 21st-40th percentile was related to higher odds of incurring dental costs <4 years of age (aOR 1.16 (1.02-1.32)) compared to the lowest percentile (Table 2).

**Table 2:** Dental costs versus no dental costs <4 years of age and subsequent dental costs versus ≥2 years no dental costs incurred by 4-year-old children and associations with child and parent variables.

	Dental costs versus no dental costs <4 years of age			Subsequent dental costs versus ≥2 years no dental costs incurred		
	Crude (Univariable logistic regression)	Adjusted (Multivariable logistic regression)		Crude (Univariable logistic regression)	Adjusted (Multivariable logistic regression)	
	**OR**	**OR**	***P***	**OR**	**OR**	***P***
**Child variables**						
**Sex**						
Male	reference	reference		reference	reference	
Female	1.04	1.04 (0.95-1.13)	.386	1.17	1.15 (1.03-1.28)	**.009**
**Migration background**						
No migration background	reference	reference		reference	reference	
Western migration background	0.77	0.76 (0.66-0.88)	**<.001**	0.84	0.83 (0.70-0.97)	**.022**
Non-Western migration background	0.89	0.94 (0.84-1.05)	.247	1.40	1.39 (1.21-1.59)	**<.001**
**Type of household**						
Couple of parents	reference	reference		reference	reference	
Single parent	0.67	0.69 (0.62-0.77)	**<.001**	0.71	0.70 (0.61-0.80)	**<.001**
Unknown/other	0.54	0.59 (0.37-0.94)	**.025**	0.49	0.56 (0.33-0.93)	**.026**
**Parent variables**						
**Household income (percentile)**						
0-20	reference	reference		reference	reference	
21-40	1.21	1.16 (1.02-1.32)	**.028**	1.19	1.18 (1.00-1.40)	.055
41-60	1.15	1.05 (0.91-1.22)	.507	0.97	0.97 (0.81-1.16)	.717
61-80	1.30	1.13 (0.97-1.32)	.129	1.04	1.03 (0.84-1.25)	.800
81-100	1.23	1.04 (0.90-1.21)	.595	0.81	0.80 (0.67-0.96)	**.014**
Unknown/other	0.61	0.65 (0.45-0.94)	**.022**	0.43	0.51 (0.34-0.76)	**.001**
**Educational level parent**						
Low	reference	reference		reference	reference	
Medium	0.91	0.87 (0.75-1.00)	0.051	.80	0.86 (0.72-1.03)	.100
High	1.17	1.02 (0.88-1.18)	0.812	.89	1.10 (0.91-1.34)	.312
Unknown/other	0.99	0.94 (0.82-1.09)	0.436	.95	0.97 (0.80-1.17)	.744

Bold values indicate statistically significance.

### Dental costs <4 years of age and consecutive dental costs and associations with (no) dental costs at age 9

Both dental costs <4 years of age (aOR 2.12 (1.83-2.45)) and consecutive dental costs (aOR 6.48 (5.56-7.54)) were significantly associated with a higher likelihood of incurring dental costs at age 9, compared to incurring no dental costs. Children with higher-educated parents and a non-Western migration background were more likely to incur dental costs at age 9, while this was less likely for children from a single-parent household (Table 3, Table 4).

**Table 3:** Dental costs versus no dental costs at age 9, in 2017 and dental costs at age 9 and associations with dental costs versus no dental costs <4 years of age for 4-year-old children, crude analyses and analyses adjusted for child and parent variables.

	Dental costs versus no dental costs at age 9			Dental costs at age 9		
	Crude (Univariable logistic regression)	Adjusted (Multivariable logistic regression)		Crude (Univariable linear regression)	Adjusted (Multivariable linear regression)	
	OR	OR	*P*	B	B	*P*
**Dental costs <4 years of age**						
No	reference	reference		reference	reference	
Yes	2.19	2.12 (1.83-2.45)	**<.001**	2.14	5.16 (-2.69-13.00)	.198
**Child variables**						
**Sex**						
Male	reference	reference		reference	reference	
Female	0.95	0.94 (0.82-1.09)	.438	−6.59	−9.74 (−17.24-−2.25)	**.011**
**Migration background**						
No migration background	reference	reference		reference	reference	
Western migration background	0.88	0.93 (0.74-1.17)	.531	34.65	24.49 (11.81-37.17)	**<.001**
Non-Western migration background	1.08	1.28 (1.06-1.53)	**.009**	101.67	61.32 (51.79-70.85)	**<.001**
**Type of household**						
Couple of parents	reference	reference		reference	reference	
Single parent	0.64	0.77 (0.64-0.92)	**.003**	13.09	−13.59 (−23.73-−3.45)	**.009**
Unknown/other	0.47	0.85 (0.42-1.70)	.642	−4.68	−35.72 (−78.77- 7.33)	.104
**Parent variables**						
**Household income (percentile)**						
0-20	reference	reference		reference	reference	
21-40	1.27	1.15 (0.92-1.44)	.223	−28.30	−14.62 (−26.09-−3.15)	**.013**
41-60	1.12	0.98 (0.77-1.25)	.880	−58.27	−23.47 (−36.39-−10.55)	**<.001**
61-80	1.29	1.06 (0.81-1.39)	.690	−88.63	−36.42 (−50.13-−22.71)	**<.001**
81-100	1.15	0.91 (0.71-1.17)	.452	−108.02	−43.18 (−56.30-−30.05)	**<.001**
Unknown/other	0.49	0.51 (0.31-0.85)	**.010**	−58.91	−30.67 (−67.84- 6.50)	.106
**Educational level parent**						
Low	reference	reference		reference	reference	
Medium	1.07	1.13 (0.90-1.43)	.294	−51.83	−31.74 (−44.60-−18.88)	**<.001**
High	1.45	1.57 (1.22-2.03)	**<.001**	−112.86	−57.31 (−70.73-−43.90)	**<.001**
Unknown/other	1.14	1.16 (0.91-1.47)	.228	−17.69	−5.32 (−18.24-7.60)	.420

Bold values indicate statistically significance.

**Table 4:** Dental costs versus no dental costs at age 9, in 2017 and dental costs at age 9 and associations with subsequent dental costs versus ≥2 years no dental costs incurred by 4-year-old children, crude analyses and analyses adjusted for child and parent variables.

	Dental costs versus no dental costs at age 9			Dental costs in at age 9		
	Crude (Univariable logistic regression)	Adjusted (Multivariable logistic regression)		Crude (Univariable linear regression)	Adjusted (Multivariable linear regression)	
	OR	OR	*P*	B	B	*P*
**Consecutive dental costs incurred (in years)**						
≥2 years no dental costs incurred	reference	reference		reference	reference	
Consecutive dental costs incurred	6.58	6.48 (5.56-7.54)	**<.001**	26.86	17.52 (7.35-27.69)	**.001**
**Child variables**						
**Sex**						
Male	reference	reference		reference	reference	
Female	0.95	0.90 (0.78-1.05)	.184	−6.59	−10.02 (−17.49-−2.55)	**.009**
**Migration background**						
No migration background	reference	reference		reference	reference	
Western migration background	0.88	0.94 (0.74-1.19)	.594	34.65	26.22 (13.65-38.79)	**<.001**
Non-Western migration background	1.08	1.13 (0.94-1.36)	.202	101.67	60.07 (50.59-69.54)	**<.001**
**Type of household**						
Couple of parents	reference	reference		reference	reference	
Single parent	0.64	0.79 (0.65-0.95)	**.011**	13.09	−13.41 (−23.51-−3.32)	**.009**
Unknown/other	0.47	0.82 (0.41-1.65)	.575	−4.68	−30.24 (−73.14-12.65)	.167
**Parent variables**						
**Household income (percentile)**						
0-20	reference	reference		reference	reference	
21-40	1.27	1.11 (0.88-1.40)	.397	−28.30	−14.10 (−25.53-−2.67)	**.016**
41-60	1.12	0.99 (0.77-1.27)	.923	−58.27	−23.10 (−35.97-−10.22)	**<.001**
61-80	1.29	1.06 (0.80-1.40)	.683	−88.63	−36.64 (−50.25-−23.02)	**<.001**
81-100	1.15	0.99 (0.76-1.28)	.935	−108.02	−41.85 (−54.90-−28.80)	**<.001**
Unknown/other	0.49	0.77 (0.44-1.34)	.358	−58.91	−32.88 (−68.34-2.58)	.069
**Educational level parent**						
Low	reference	reference		reference	reference	
Medium	1.07	1.21 (0.95-1.54)	.120	−51.83	−31.08 (−43.89-−18.27)	**<.001**
High	1.45	1.60 (1.23-2.08)	**<.001**	−112.86	−57.96 (−71.31-−44.61)	**<.001**
Unknown/other	1.14	1.16 (0.91-1.48)	.244	−17.69	−7.21 (−20.09-5.67)	.273

Bold values indicate statistically significance.

### Dental costs <4 years of age and consecutive dental costs and associations with the amount of dental costs at age 9

In the multivariable analyses, whether dental costs <4 years of age were incurred was not associated with dental costs at age 9 (mean difference (MD) in euros 5.16 (-2.69 to 13.00)). For children incurring consecutive dental costs, the dental costs were significantly higher compared to children without consecutive dental costs (MD 17.52 (7.35-27.69)). Children with a migration background and those living with a single parent were more likely to incur higher dental costs. Regarding household income, there was a significant decrease in the amount of dental costs across the higher percentiles compared to the lowest percentile. A higher parental educational level was associated with lower dental costs compared to a low educational level (Table 3, Table 4).

## Discussion

In Amsterdam, 9 out of 10 children from the age of 6 to 9 went to a dentist, with mean dental costs per year in the range of 219.96-244.66 euros. Children with a Western migration background or living with a single parent were less likely to visit a dentist early or regularly. Also, fewer children from families with a high household income went for regular dental visits. Children with a non-Western migration background were more likely to go for regular dental visits. Early and regular dental visits were significantly related to dental visits in the future. For children with a dental visit later in childhood, at age 9, the amount of dental costs was not associated with whether an early first dental visit took place. Yet, children with regular dental visits incurred significantly higher dental costs later in childhood, although the mean difference was only 17.52 euros. Having a migration background, a lower household income, and a lower parental educational level were the most influential sociodemographic characteristics.

The vast majority of children from 4 years and older went to a dentist, which suggests that access to oral health care is good in Amsterdam: either parents bring their children to a dental practice or the children are seen for a check-up at their school by a dentist from the Jeugdtandverzorging (JTV), an organization for youth dental care in Amsterdam.^31^ Probably the most important reason is that the dental costs of children are covered by the parental mandatory basic medical insurance, so there is no financial threshold for parents. However, most parents do not adhere to the recommendation to bring their child to a dentist when the first tooth erupts,^4^^,^^16^ possibly caused by a lack of knowledge about oral health and the importance of an early first dental visit.^32^ Also, it should be taken into account that the recommendation for a first dental visit around the eruption of the first teeth was not yet in force in 2009 in the Netherlands.

Early and regular dental visits were significantly associated with visiting a dentist years later in childhood. This is in line with several other studies that have shown that early (preventive) dental visits and regular dental visits in the following years are related to regular dental attendance and better oral health in the future. The rationale is that parents who bring their children to a dentist for regular check-ups are more motivated and that they receive timely guidance to provide good daily oral hygiene and nutrition in the home setting.^4^^,^^33^^,^^34^ However, this was put into perspective by Beil et al., who stated that early preventive treatments are mostly efficient in children with high caries risk and much less in children with low(er) risk of developing caries. For the latter group, it is less urgent to visit a dentist before the age of 3 because early and regular dental visits were not significantly related to better oral health later in childhood.^35^^,^^36^

For children with dental visits later in childhood, the amount of dental costs, and thus the volume of oral health care consumption, was slightly higher for those with early and regular dental visits. This is in disagreement with the expectation that early and regular dental visits contribute to less oral health care consumption in the following years, since early preventive dental visits are associated with a reduced restorative treatment need in the future.^33^^,^^34^^,^^37^^,^^38^ More (instead of less) oral health care consumption for children with early and regular dental visits could be caused by a large volume of preventive oral health care throughout childhood to prevent dental caries, possibly applied to both children with high risk and low risk for dental caries, and the latter receiving more preventive care than needed.^39^ Additionally, dental caries develops over time, and high-risk children may have more extensive dental treatment needs only years later during adolescence.^40^^,^^41^ That could explain our results that the volume of oral health care consumption slightly differed between the children with and without early and regular dental visits, since there was a relatively short follow-up.

Low(er) household income, low(er) parental educational level, and having a migration background seemed to moderately influence early and regular dental visits by young children in Amsterdam. But for children with dental visits later in childhood, those sociodemographic determinants were strongly associated with incurring higher dental costs, most likely due to a higher prevalence of dental caries and thus more (restorative) treatment, which is in agreement with previous studies.^3^^,^^27^^,^^42^^,^^43^ The social gradient in caries experience that is already present in early childhood diverges in adulthood. Verlinden et al. showed that among 23-year-olds who had dental caries during childhood, those from families with a low SEP experienced significantly more caries than their peers, highlighting the importance of preventing caries development at an early age.^15^

A major strength of this study was that a very complete dataset with a large sample of all 4-year-old children living in Amsterdam was analysed, obtained from a trustworthy source with longitudinal data, which allowed insight into dental visits over a 9-year time span. A limitation was that no clinical oral health outcomes and no dental performance codes were available. Data on dental costs from Statistics Netherlands were analysed as a proxy variable. Therefore, the results could not be specified into the type of oral health care provided, and no conclusions could be drawn about possible associations between the provided oral health care and oral health status. Also, since the follow-up was only 5 years, covering the first part of childhood, no conclusions can be drawn about oral health care patterns during adolescence. Hence, future research should aim to study patterns of oral health care consumption from early childhood to late adolescence by extending the follow-up period to, preferably, at least 10 years. Dental performance codes are necessary to specify the type of oral health care provided, and to assess possible associations with oral health status, future research should include clinical outcomes, such as caries experience. Regarding geographical limitations, this research was conducted in Amsterdam, which could affect the generalizability, since the sociodemographic characteristics are different from those in the rest of the country, and thus the results cannot be extrapolated.^44^ Future research could be extended to children residing in other locales in the Netherlands.

To conclude, sociodemographic characteristics such as migration background, living with a single parent, and household income moderately contributed to whether an early first dental visit and regular dental visits took place. Early and regular dental visits were significantly related to dental visits (versus no dental visits) later in childhood. Yet, for dental visits that took place later in childhood, children with early and regular visits incurred higher dental costs than those without. Therefore, from a health economic perspective, it cannot be concluded that a first dental visit at a young age or regular dental visits in childhood lead to lower dental costs later in life. No general conclusions can be drawn from this study on the possible effects and benefits of early and regular dental visits on children’s oral health over their life course. Further research is needed to identify the patterns of oral health care consumption and their association with oral health status during childhood and to extend the follow-up into adolescence.
